# Enzymatic properties of *Staphylococcus aureus *adenosine synthase (AdsA)

**DOI:** 10.1186/1471-2091-12-56

**Published:** 2011-10-28

**Authors:** Vilasack Thammavongsa, Olaf Schneewind, Dominique M Missiakas

**Affiliations:** 1 Department of Microbiology, University of Chicago, 920 E. 58th St., Chicago, Illinois 60637, USA

## Abstract

**Background:**

*Staphylococcus aureus *is a human pathogen that produces extracellular adenosine to evade clearance by the host immune system, an activity attributed to the 5'-nucleotidase activity of adenosine synthase (AdsA). In mammals, conversion of adenosine triphosphate to adenosine is catalyzed in a two-step process: ecto-nucleoside triphosphate diphosphohydrolases (ecto-NTDPases) hydrolyze ATP and ADP to AMP, whereas 5'-nucleotidases hydrolyze AMP to adenosine. NTPDases harbor apyrase conserved regions (ACRs) that are critical for activity.

**Results:**

NTPDase ACR motifs are absent in AdsA, yet we report here that recombinant AdsA hydrolyzes ADP and ATP in addition to AMP. Competition assays suggest that hydrolysis occurs following binding of all three substrates at a unique site. Alanine substitution of two amino acids, aspartic acid 127 and histidine 196 within the 5'-nucleotidase signature sequence, leads to reduced AMP or ADP hydrolysis but does not affect the binding of these substrates.

**Conclusion:**

Collectively, these results provide insight into the unique ability of AdsA to produce adenosine through the consecutive hydrolysis of ATP, ADP and AMP, thereby endowing *S. aureus *with the ability to modulate host immune responses.

## Background

*Staphylococcus aureus *is a Gram-positive pathogen and the leading cause of bloodstream, lower respiratory tract, skin and soft tissue infections [1]. *S. aureus *produces numerous virulence factors that contribute to its ability to cause disease [2-4]. These include several toxins that are known for their detrimental effects on host cells [5,6], in particular cells of the immune system [7,8]. Staphylococci can infect a broad range of tissues and organs resulting in excessive tissue damage [9]. This observation is highlighted by the appearance of large populations of necrotic cells surrounding staphylococcal communities within organ abscesses isolated from infected mice [10]. Cellular damage caused by bacterial triggers the release of otherwise sequestered intracellular components such as heat shock proteins (HSPs) [11], S100 proteins [12], nucleosomes [13], N-formylated mitochondrial peptides [14] and purines (ATP and ADP) [15,16] all of which are known to potently stimulate inflammation. Excessive inflammation can be detrimental to the host due to the prolonged presence of activated immune cells as well as the leakage of proteases and other noxious agents that damage surrounding tissues. A delicate balance of pro- and anti-inflammatory mediators is critical to prevent extensive inflammation.

Extracellular nucleotides (i.e. adenosine tri-, di- and monophosphates and adenosine), which signal through purinergic cell surface receptors have recently been shown to serve as mediators of inflammation. For example, stimulation of purinergic PY receptors by ATP and ADP results in pro-inflammatory responses while stimulation of PX adenosine receptors leads to an anti-inflammatory response [17-22]. In addition, nucleotide metabolizing enzymes that hydrolyze adenosine tri- and di-phosphates (ATP and ADP) or adenosine monophosphates (AMP), termed ecto-nucleoside triphosphate diphosphohydrolases (ecto-NTPDases) or 5'-nucleotidases respectively, regulate purinergic signaling by controlling the level of extracellular nucleotides. NTPDases hydrolyze nucleoside tri- and/or diphosphates, but not monophosphates [23-25]. Eight members of the NTPDase family have been identified in mammals, all of which are characterized by five highly conserved sequence motifs known as "apyrase conserved regions" (ACR), which range from 4-13 residues in length [26]. CD39 (NTDPase 1) was the first member identified for this family of enzymes. It is expressed on activated B cells and regulatory T (T_reg_) cells. Hydrolysis of 5'-AMP is carried out by a second class of enzymes. CD73 is the best characterized of the 5'-nucleotidases; CD73 hydrolyzes 5'-AMP specifically and shows no activity towards 2'- and 3'-monophosphates [27]. This ecto-enzyme is expressed in different tissues, with abundant expression in the colon, kidney, liver, heart, lung and on specific cells of the immune system [27,28]. CD73 and CD39 are co-expressed on the surfaces of CD^+4^/CD^+25^/Foxp3^+ ^T_reg _cells and catalyze the enzymatic conversion of ATP/ADP-derived AMP into the anti-inflammatory mediator adenosine, subsequently leading to inhibition of T cell proliferation and secretion of cytokines [29,30].

We recently reported that *S. aureus *AdsA, a cell wall anchored protein, is a 5'-nucleotidase that catalyzes the conversion of AMP to adenosine. The nucleotidase activity of AdsA is critical for *S. aureus *survival in blood and *adsA *mutants are impaired in their ability to induce abscess formation during infection [31]. Thus, we surmise that *S. aureus *uses AdsA to increase the concentration of adenosine concentrations within the host and take advantage of adenosine's immunosuppressive properties to escape immune clearance. Since staphylococci are surrounded by large populations of dead or dying host cells within deep tissue abscesses [10], it can be assumed that there is an abundance of extracellular nucleotides released from damaged tissues. The importance of extracellular nucleotide signaling in mediating pathogen clearance led us to further investigate AdsA's nucleotide metabolizing capacity. Although analyses of the amino acid sequence of AdsA did not reveal conserved ACR motifs indicative of NTPDases, a recombinant AdsA was able to efficiently hydrolyze both ATP and ADP *in vitro*. We further characterized the enzyme kinetics of AdsA hydrolysis of ATP, ADP and AMP and also identified amino acid residues critical for AdsA's hydrolase activity.

## Methods

### Purification of recombinant AdsA

Recombinant GST-tagged AdsA (rAdsA) was expressed using pVT1 in *Escherichia coli *BL21 (DE3) and purified using glutathione S-transferase affinity chromatography as described previously [31]. The N-terminal GST tag was cleaved with thrombin and thrombin removed by incubation with benzamadine sepharose beads per manufacturer's conditions (GE Healthcare).

### Assays for enzymatic activity of AdsA

Hydrolysis of ATP, ADP and AMP (Sigma-Aldrich) was carried out in 50 mM Tris-HCl buffer pH 7.5, in the presence of 1 mM nucleotide and 0.5 mM MnCl_2_. rAdsA was added to the reaction at 0.15 μg/μl and the reaction was incubated at 37°C for 15 min. Inorganic phosphate release was detected by addition of malachite green dye reagent [32] (1.1% w/v ammonium molybdate, 0.04% w/v malachite green hydrochloride) and 3.4% citric acid and concentration was calculated from a known concentration range of phosphate standards. Similar conditions were used to determine the pH optima of rAdsA for AMP and ADP. Inorganic phosphate release was also recorded using malachite green dye reagent to assess hydrolysis of non-adenine based nucleotides. To determine the divalent cation preference of rAdsA, ADP hydrolysis (1 mM) was assayed in 50 mM Tris-HCL buffer pH 7.5, containing either 0-5 mM MgCl_2 _or MnCl_2_, or 0-2.5 mM ZnCl_2_, or CuSO_4_. Thin layer chromatography was performed as previously described [31]. Purified rAdsA (2 μM) was incubated in a 15 μl reaction volume with increasing amounts of [^14^C]AMP (Moravek biochemicals) in 50 mM Tris-HCL buffer, pH 7.5 containing 0.5 M sucrose and 0.5 mM MnCl_2_. Samples were incubated for 15 minutes and then spotted onto silica plates, followed by separation by TLC using a 75:25 isopropanol/double distilled H_2_O-0.2 M ammonia bicarbonate solvent. 75 μM cold nucleotide (AMP or ADP) was used for competitive inhibition experiments.

### Site-directed mutagenesis of rAdsA

The following primers were used for PCR amplification reactions:

D127F (5'-ACAACACATAAAATATTACA TACAAATGCTATCCATGGCCGACTAGC-3'), D127R (5'-GCTAGTCGGCCATGGATA GCATTTGTATGTAATATTTTATGTGTTGT-3'), H129F (5'-ACACATAAAATATTACATACAAATGATATCGCTGGCCGACTAGCCGAAG A-3'), H129R (5'-TCTTCGGCTAGTCGGCCAGCGATATCATTTGTATGTAATATTTTAT GTGT-3'), H196F (5'-GATG CTATGGCAGTCGGTAACGCTGAATTTGACTTTGGATAC-3'), H196R (5'-GTATCCA AAGTCAAATTCAGCGTTACCGACTGCCATAGCATC-3'), D199F (5'-GTCGGTAAC CATGAATTTGCCTTTGGATACGATCAGTTG-3'), D199R (5'-CAACTGATCGTATC CAAAGGCAAATTCATGGTTACCGAC-3'). Site-directed mutagenesis was performed using AccuPrime *pfx *DNA polymerase (Invitrogen) using pVT1 as a template for replacement of Asp^127 ^to Ala (D127A), His^129 ^to Ala (H129A), His^196 ^to Ala (H196A), and Asp^199 ^to Ala (D199A). All plasmids were transformed in *E. coli *BL21 (DE3) to produce recombinant variants. All mutations were confirmed by nucleotide sequencing of plasmid DNA.

### Assessment of nucleotide binding to rAdsA

Binding of AMP and ADP to rAdsA was carried out as described [33]. Briefly, rAdsA (10 μM) was incubated with 9 μCi [^14^C]AMP or 9 μCi [^14^C]ADP for 15 min on ice in 50 mM Tris-HCl buffer, pH 7.5 containing 0.5 M MnCl_2_. Samples were adsorbed to a nitrocellulose membrane using a vacuum manifold and washed twice with 10 ml buffer. Radioactivity retained on the membranes was measured by scintillation counting.

### Circular dichroism

rAdsA was dialyzed against 8 mM NaH_2_PO_4_, 1.5 mM Na_2_HPO_4 _buffer. Purified protein (100 μg/ml) was subjected to circular dichroïsm using an AVIV 202 CD spectrometer at room temperature.

## Results

### AdsA hydrolyzes adenosine nucleoside tri- and di-phosphates

**Figure 1:**
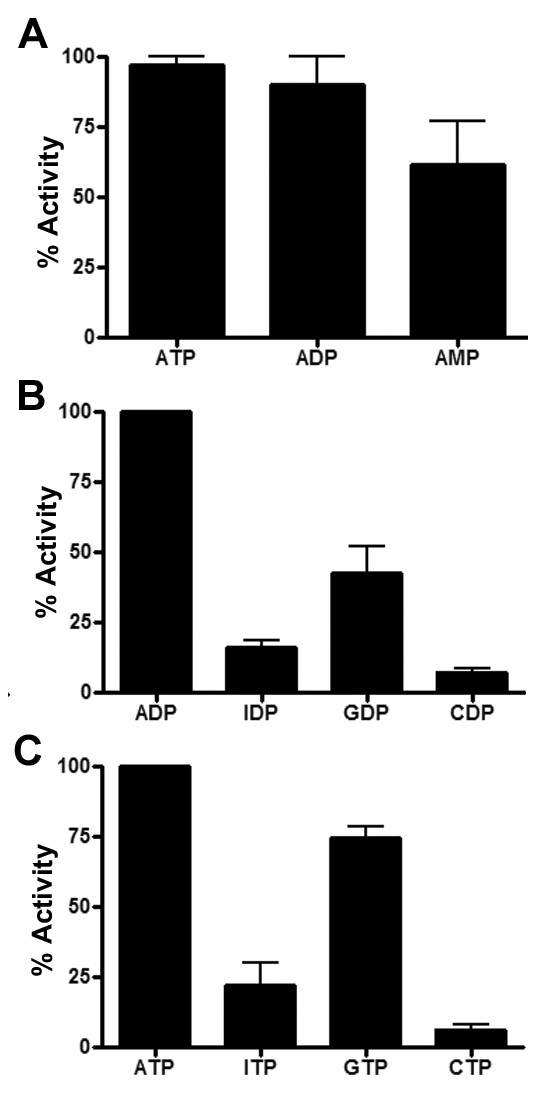
**AdsA efficiently hydrolyzes adenosine nucleoside tri- and diphosphates**. The ability of rAdsA to hydrolyze nucleoside tri- or diphosphates was evaluated by assessing inorganic phosphate released after incubation of rAdsA with the indicated substrates for 15 minutes. Percent activity is expressed as the amount of inorganic phosphate released relative to ATP (A and C, 914 ± 70 nmol pi) and ADP (B, 840 ± 55 nmol pi) within each experiment. The results are the average of 3 independent analyses conducted in duplicate and the error bars represent the SEM.

We have previously shown that *S. aureus *AdsA hydrolyzes AMP to produce adenosine [31]. However, extracellular ATP and ADP stimulate inflammation as well. We wondered whether AdsA might also hydrolyze these molecules. Hydrolysis was assessed following incubation with rAdsA for 15 min by measuring the release of inorganic phosphate using the malachite green colorimetric assay. rAdsA hydrolyzed ATP and ADP efficiently. In fact, the enzyme released 1.5 fold more phosphate from ATP and ADP after 15 min incubation than from AMP (Figure 1A). In addition, rAdsA hydrolyzed both guanosine derivatives GTP and GDP, albeit at reduced efficiency compared to ATP and ADP. In comparison, the inosine nucleosides ITP and IDP were poor substrates for the enzyme (Figure 1B, C). Cytosine nucleosides were not hydrolyzed by rAdsA (Figure 1B, C).

In mammals, nucleotide di- and tri-phosphate hydrolysis is primarily attributed to NTPDases whereas 5'-nucleotidases display specificity toward nucleotide mono-phosphate substrates. NTPDases encompass five conserved ACR motifs that form the active site of these enzymes. Such ACR motifs indicative of NTPDases cannot be found in the primary sequence of AdsA. NTPDases are rarely found in prokaryotes, however bacterial 5'-nucleotidases from *Escherichia coli *and *Vibrio costicola *have been shown to possess the capacity to hydrolyze ATP molecules [34,35]. Thus, our results suggest that the bacterial 5'-nucleotidase AdsA utilizes a distinctive mechanism for the hydrolysis of ADP/ATP that has been shown to occur in mammalian NTPDases.

### Kinetic activity of AdsA

**Figure 2:**
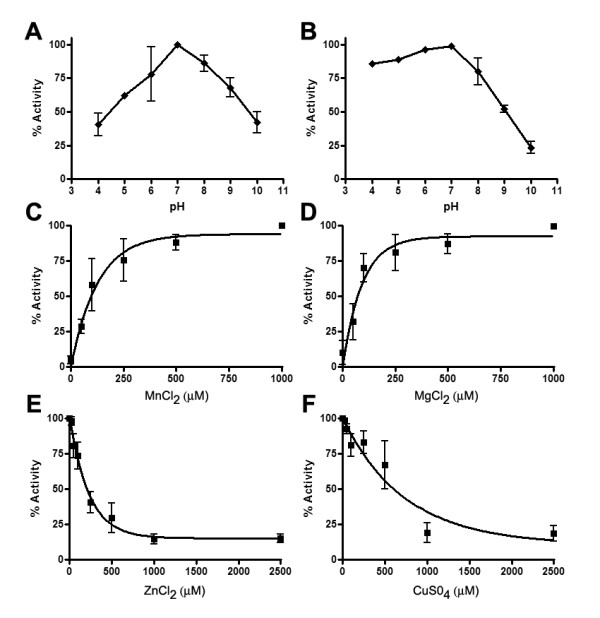
**Kinetic activity of AdsA**. (A) hydrolysis of 1 mM AMP, 1 mM ADP and 1 mM ATP by 0.15 μg/μl rAdsA, measured as nmol Pi released over time. The results are expressed as nmol Pi released over time and the average of two independent analyses conducted in triplicate. Error bars represent the standard error of the mean. (B) Michaelis-Menten plots of enzyme velocity demonstrating the effect of varying AMP (upper panel) or ADP (lower panel) concentration as shown. Curve fitting using nonlinear regression was performed using GraphPad Prism5 software. The results are expressed as average μmol Pi released/min/mg protein and error bars represent the standard error of the mean. Data is representative of two independent analyses conducted in triplicate.

Substrate preference for nucleoside di- and tri-phosphates varies among members of the ecto-NTPDase family of enzymes. For example, CD39/NTPDase1 hydrolyzes both ATP and ADP with similar efficiency, whereas NTPDase2 preferentially exhibits ATPase activity [26]. We examined and compared the relative rate of ATP, ADP and AMP hydrolysis by rAdsA at pH 7.5 in the presence of 0.5 mM MnCl_2 _(Figure 2A). The rate of inorganic phosphate released from all three nucleotides was linear over the first 20 min of incubation (with linear regression using GraphPad Prism5 software yielding r^2 ^value of 0.90, 0.98 and 0.96 for AMP, ADP and ATP hydrolysis, respectively). The initial rate of reaction varied with the concentration of substrate (AMP or ADP) and followed the Michaelis-Menten kinetic model of a single-substrate reaction (Figure 2B). GraphPad Prism5 software was used to perform nonlinear regression analysis and a *V_max _*value 713 μmol Pi/min/mg for ADP hydrolysis compared to 391 μmol Pi/min/mg for AMP hydrolysis together with a calculated *K_m _*value of 0.93 mM for AMP and 0.51 mM for ADP. The total release of inorganic phosphate from 1 mM ADP was approximately 1.8 times higher than from 1 mM AMP after 10 minutes (Figure 2B). ADP contains two phosphate groups to a single phosphate group in AMP suggesting similar hydrolysis rates for both the α- and β-phosphate groups. However, only a slight increase in inorganic phosphate was observed from 1 mM ATP relative to 1 mM ADP over the same time period, suggesting that AdsA may preferentially hydrolyze the α- and β-phosphate groups of AMP and ADP compared to the γ-phosphate in ATP. Together these results confirm that AdsA is capable of hydrolyzing ATP and ADP in addition to its known AMP substrate.

### Effect of pH and metal cations on AdsA activity

**Figure 3:**
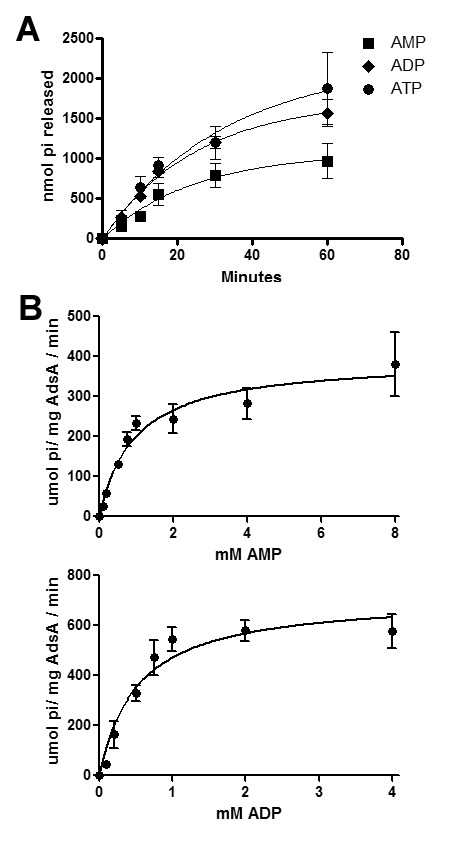
**AdsA is highly active at a near neutral pH and is dependent on metal cations**. (A and B) Effect of pH on activity of rAdsA. Relative hydrolysis of 1 mm AMP (A) or 1 mm ADP (B) in buffer containing 50 mm Tris-HCl and 0.5 mM MnCl_2 _over the indicated pH range is shown. The results are expressed as the percentages of activity and are the average of three independent analyses. (C-D), Shown is the relative ADPase activity of AdsA in the presence of (C) MnCl_2_, (D) MgCl_2_, (E) ZnCl_2_, and (D) CuCl_2_. The results are expressed as relative activity of the highest value in each experiment. Data is the average of two independent analyses conducted in triplicate.

Increased concentrations of adenine nucleotides in the extracellular milieu may contribute to the drop in pH observed during severe inflammation and hypoxia. This prompted us to evaluate the effect of pH on both ADP and AMP hydrolysis over a pH range of 4-10. AdsA displayed optimal activity at pH 7.0 when AMP and ADP served as substrates. However, substantial ADPase activity was observed as low as pH 4.0 (Figure 3A, B). This finding suggests that the enzyme remains active in various acidic environments that bacteria encounter during host invasion.

All mammalian surface-located NTPDases are inactive in the absence of Mg^2+ ^or Ca^2+ ^cations [26]. In contrast, the majority of parasitic enzymes are stimulated by Mg^2+ ^or Ca^2+ ^[36-38] and Zn^2+^. Hydrolysis of ATP and ADP by other bacterial 5'-nucleotidases require Mg^2+ ^or Mn^2+ ^and these enzymes are inhibited by Zn^2+ ^[34]. We have previously shown that optimum hydrolysis of AMP by staphylococcal AdsA is stimulated by Mg^2+ ^and Mn^2+ ^and inhibited by Zn^2+ ^and Cu^2+ ^[31]. Here, we examine how divalent cations modulate the ADPase activity of rAdsA. Similar to AMP hydrolysis, we observe that optimal ADPase activity occurred in the presence of Mn^2+ ^and Mg^2+ ^specifically at 0.5 mM MnCl_2 _and MgCl_2 _(Figure 3C, D). The presence of Zn^2+ ^and Cu^2+ ^inhibited hydrolysis of ADP. The 50% Inhibitory Concentration (IC_50_) for Zn^2+ ^and Cu^2+ ^were calculated using non-linear regression as 159 μM and 512 μM, respectively (Figure 3E, F). These results suggest that hydrolysis of AMP and ADP may require similar catalytic reactions and active site residues.

### Inhibition of AdsA 5'-nucleotidase activity by ADP

**Figure 4:**
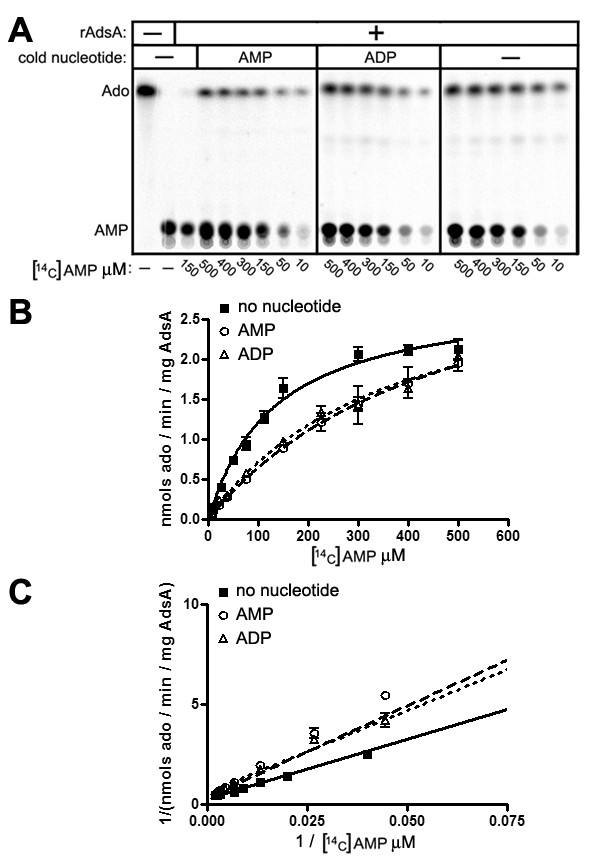
**Inhibition of AdsA 5'-nucleotidase activity by ADP**. 2 μM rAdsA was incubated with 0-500 μM [^14^C]AMP in the presence of 75 μM cold AMP or ADP as indicated. (A) Radioactive signals for [^14^C]AMP and [^14^C]Ado after TLC were captured by a phosphorimager. Data is representative of two independent analyses. (B) Curve fitting analysis of data from (A) using nonlinear regression was performed using GraphPad Prism5 software. The results are expressed as nmols [^14^C]adenosine produced/min/mg rAdsA and are the averages of two experiments performed in duplicate. (C) Lineweaver-Burk plot of data in (B).

The crystal structure of *S. aureus *AdsA has not been determined and the molecular interactions between AdsA and its substrates are unknown. To examine AdsA's substrate interactions further, we asked whether ADP is a competitive inhibitor of rAdsA's 5'-nucleotidase activity. rAdsA was incubated with [^14^C]AMP and increasing concentrations of cold non-radiolabeled AMP or ADP and production of [^14^C]adenosine was measured by separation of substrate and product using thin layer chromatography (TLC) and recording of radioactive counts. (Figure 4A) and the data were analyzed using the non-linear curve fitting of the GraphPad Prism software to determine *K_m _*values (Figure 4B). Co-incubation of either cold AMP or ADP with [^14^C]AMP similarly inhibited the hydrolysis of [^14^C]AMP, as the *K_m _*value of [^14^C]AMP in the presence of either AMP or ADP was reduced to similar levels. Furthermore, Lineweaver-Burk analyses (Figure 4C) clearly show that cold AMP and ADP function as similar competitive inhibitors of the reaction, suggesting that ADP and AMP may compete for the same AdsA substrate binding site.

### Residues in the 5'-nucleotidase signature sequence that contribute to AdsA activity

Extensive site-directed mutagenesis studies have been carried out with both CD39/NTPDase1 and NTPDase3 [39-42], as well as bacterial 5'-nucleotidases [27,43,44] to reveal the importance of conserved residues to catalytic activities. The structure-function analysis of *E. coli *5'-nucleotidase (UDP-sugar hydrolase) identified residues within its first nucleotidase signature sequence that are implicated in binding divalent metal cations. This analysis also revealed that the enzyme's catalytic Asp-His dyad is located in the second nucleotidase signature sequence. Similar amino acid residues and signature sequences can be identified in the primary sequence of staphylococcal AdsA. Specifically, Asp^127 ^and His^129 ^are located in the first signature sequence, ILHTnD^127^iH^129^GrL, whereas His^196 ^and Asp^199 ^are located in the second signature sequence, YdamaVGNH^196^EFD^199^. To examine the contribution of these four amino acids to AdsA catalysis, we individually substituted Asp^127^, His^129^, His^196 ^and Asp^199 ^for alanine. Each variant rAdsA was purified and its catalytic activity and substrate specificity was compared to that of the wild-type enzyme.

**Figure 5:**
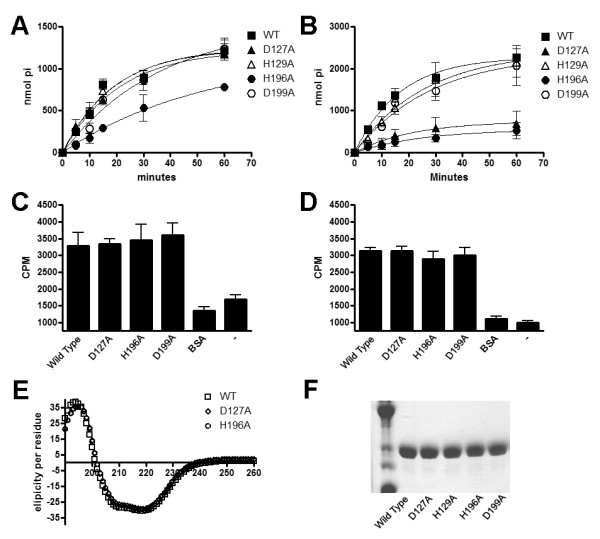
**Amino acid substitutions of conserved residues present in the 5'-nucleotidase signature sequences affects the level of AdsA activity**. (A and B) hydrolysis of 1 mm AMP (A) or 1 mM ADP (B) by 0.15 μg/μl rAdsA measured as nmol P_i _released over time. (C and D) Binding of AMP or ADP to rAdsA protein. Protein in mixtures of rAdsA and [^14^C]AMP (C) or [^14^C]ADP (D) were adsorbed onto nitrocellulose filters and the amount of radioactivity retained on the filter was determined. The results are expressed as radioactive counts per minute (cpm). Data is representative of two independent analyses conducted in triplicate and error bars represent the SEM. (E) CD spectra of WT rAdsA, rAdsAD127A and rAdsAH196A. (F) SDS-PAGE analyses of 5 μg protein as quantified by a BCA protein assay. Lanes 1-5 correspond to WT, D127A, H129A, H196A and D199A respectively.

The release of inorganic phosphate was monitored when using either AMP or ADP as substrates. Replacing His^129 ^or Asp^199 ^with alanine did not affect the ability of the enzyme to hydrolyze AMP or ADP. However, substitution of Asp^127 ^to Ala reduced ADP hydrolysis to 30% ± 4% of wild type levels but did not affect the ability of rAdsA to hydrolyze AMP. Conversely, subsitution of His^196 ^to Ala led to a moderate reduction of AMP hydrolysis (65% ± 3% of wild-type activity) but significantly affected the ADPase activity of the enzyme (23% ± 5% of wild-type activity) (Figure 5A,B). Next, we asked whether reduced AMP or ADP hydrolysis by the variant proteins (specifically Asp^127^Ala, His^196^Ala and Asp^199^Ala) could be explained by a loss of affinity for the substrates. Wild-type and variants of rAdsA were incubated with either [^14^C]AMP or [^14^C]ADP at 4°C degrees for 10 min and adsorbed onto a nitrocellulose membrane. Unbound [^14^C] labeled nucleotide was removed by extensive washes of the membranes and measurements of radioactivity counts were recorded as a direct assessment of nucleotide binding to the enzymes. All three mutants (Asp^127^Ala, His^196^Ala and Asp^199^Ala) bound [^14^C]AMP or [^14^C]ADP with affinities similar to the wild type rAdsA enzyme (Figure 5C,D). Furthermore, we recorded circular dichroism (CD) spectra and confirmed that none of the substitutions altered the secondary structure of rAdsA (Figure 5E). Together, these data identify rAdsA residues implicated in metal ion binding as well as its Asp-His catalytic dyad, two AdsA features that are shared with other 5'-nucleotidases.

## Discussion

We have previously shown that AdsA secreted by *S. aureus *hydrolyzes AMP to produce adenosine, which enhances the ability of *S. aureus *to evade immune clearance [31]. In this study, we defined the enzymatic properties of rAdsA and demonstrated that in addition to exhibiting 5'-nucleotidase activity, rAdsA also exhibits NTPDase activity. This was exemplified by rAdsA's ability to hydrolyze ADP and ATP and to a lower extent GDP and GTP, with its ADPase activity retained even under acidic conditions. In contrast, the enzyme was not able to utilize CDP or CTP.

In mammals, the conversion of ATP to adenosine requires the sequential activity of ecto-NTPDases and 5'-nucleotidases. The substrate specificities of the two types of enzyme are quite specific as CD39 (NTPDase1) cleaves ATP and ADP but not AMP [26] and likewise CD73 (5'-nucleotidase) cleaves AMP but not ATP and ADP. Substrate specificity is thought to result from structural differences between the binding pockets of NTPDases and 5'-nucleotidases. Active site residues lying within the NTPDase ACR motifs are shown to be situated in close proximity of the γ- and β-phosphate groups of ATP whereas the α-phosphate of an AMP molecule would be further buried and inaccessible. We identified amino acid residues Asp^127 ^and His^196 ^within the conserved 5'-nucleotidase signature sequences as being critical for ADP hydrolysis. Furthermore, results from the competitive inhibition experiments with cold nucleotide substrates displayed in Figure 4 imply that AdsA binds AMP and ADP at a single site. Together these observations suggest that the two 5'-nucleotidase signature sequences of AdsA lie within close proximity of the nucleotide binding pocket in a unique spacial orientation that allows for the removal of both the β- and α-phosphates. Comparison of crystal structures from AdsA bound to AMP or ADP substrates is needed to further our understanding of AdsA's unique enzymatic activity; this is currently being pursued in the laboratory.

The ability to produce adenosine from multiple substrates provides a clear advantage for *S. aureus *in the fight against the host's immune system. Initiation of staphylococcal infections usually involve bacterial invasion of the skin or blood stream via trauma, surgical wounds, or medical devices [45] and much is known about the mechanisms that *S. aureus *uses to combat the initial innate immune defense in the blood. Advanced *S. aureus *infection leads to dissemination of staphylococci into various tissues and formation of abscesses in organs. However, the molecular mechanisms of abscess formation during staphylococcal infections are not clearly understood but likely involve both pathogen and host response factors [46]. The architecture of kidney abscesses observed in cross sections of kidneys collected from mice 5 days after staphylococcal infection shows a central staphylococci community surrounded by several distinct layers of infiltrating immune cells [10]. Closer examination of the abscesses in the histological images reveals a population of necrotic immune cells directly surrounding foci of bacteria, which likely encompasses a localized environment rich in cellular debris. As high concentrations of nucleotides are likely available as substrates for AdsA, *S. aureus *may be able to increase the abundance of adenosine to local concentrations that are even higher than those observed in blood [31]. In turn, the accumulation of adenosine may diminish the bactericidal attributes of infiltrating immune cells or alter the spectrum of immune cells that arrive at the abscess lesions. Furthermore, it has been shown that inflammatory microenvironments are significantly more acidic due to hypoxia and high levels of adenosine. AdsA's ADPase activity is retained at low pH (Figure 3B), an attribute that may be important in pathogenesis under these conditions. Consistent with these observations, we have shown that kidneys isolated from mice infected with wild-type *S. aureus *harbored higher number of abscesses as compared to mice infected with isogenic *adsA *variants [31].

*S. aureus *is known to survive within the host for prolonged periods of time, however the mechanisms involved in such a lifestyle are not clearly known [reviewed in [47,48]]. The conversion of GTP to GDP inside cells is critical to intracellular signaling events [49] and the ability of staphylococci to hydrolyze GTP substrates may play a role in host intracellular survival, an area of investigation that we believe warrants further examination.

## Conclusions

We show that in addition to its 5'-nucleotidase function, AdsA is functionally similar to NTPDase enzymes, owing to its ability to hydrolyze both ATP and ADP substrates. AdsA does not harbor any NTPDase ACR motifs and suggests that bacterial 5'-nucleotidases such as AdsA may harbor a unique active site. Comparative structural analyses between AdsA and NTPDases may enable the future design of inhibitors that block not only *S. aureus *AdsA but perhaps even the AdsA homologs from other bacterial pathogens [31].

## Abbreviations

AdsA: adenosine synthase A; NTPDase: nucleoside triphosphate diphosphorylase; ATP: adenosine triphosphate; ADP: adenosine diphosphate; AMP: adenosine monophosphate; Ado: adenosine; ACR: apyrase conserved regions; CD: cluster of differention; HSP: heat shock protein; TLC: thin layer chromatography; PCR: polymerase chain reaction; CD: circular dichroism; GTP: guanosine triphosphate; GDP: guanosine diphosphate; ITP: inosine triphosphate; IDP: inosine diphosphate.

## Authors' contributions

VT defined the concept and conducted the experiments of the study. VT, OS, and DM drafted the manuscript. All authors have read and approved the final manuscript.
